# Bilateral pectoralis advancement flaps and transverse plate fixation system for sternal reconstruction in the complicated sternal wound

**DOI:** 10.1186/1749-8090-8-S1-P57

**Published:** 2013-09-11

**Authors:** JM De Raet, H De Praetere, PT Sergeant

**Affiliations:** 1 Department of Cardiac Surgery, Heart Center Leipzig, University of Leipzig, Leipzig, Germany; 2 Department of Cardiac Surgery, University Hospitals Leuven, Leuven, Belgium

## Background

Complicated sternal wound infection after cardiac surgery has an incidence of 0.4 – 6.9 % and mortality of 7 – 80 %. Aim was to report our experience with bilateral pectoralis advancement flaps and a transverse plate fixation system for sternal reconstruction in the complicated sternal wound.

## Methods

Between 2008 and 2012, 24 patients with a complicated sternal wound underwent a sternal reconstruction with bilateral pectoralis advancement flaps and a transverse plate fixation system. The median age of the cohort (4 female and 20 males), was 65.8 years (range: 33-83 years). In 19 patients, a bilateral internal thoracic artery had been used. Considerable preoperative risk factors were present: morbid obesity with Body Mass Index (BMI) ≥ 35 (range: 35 – 49.7: 13 patients); chronic obstructive pulmonary disease (COPD) without steroid therapy preoperatively (7 patients); diabetes mellitus (7 patients). Concomitant laparoscopically harvested omentoplasty was performed in case of overt mediastinits (4 patients). In 14 cases, the mediastinal wound was prepared with negative pressure wound therapy following surgical debridement. An internal fixation of the sternum by titanium locking plates with sternal and rib-to-rib fixation and bilateral pectoralis advancement flaps were performed in all patients. The postoperative course of was followed by clinical follow-up.

## Results

Early postoperative sternal stability was seen in all 24 patients. The 30-day perioperative mortality rate was zero, with an overall survival of 100% until today. Postoperatively 2 (8.3%) small superficial and 1 (4.1%) deep surgical site infection (SSI) were appreciated. Follow-up ranged from 6.5 to 54.5 months (median: 26.5 months).

## Conclusions

Combination of bilateral pectoralis advancement flaps and a transverse plate fixation system for sternal reconstruction can contribute to a successful outcome following a complicated sternal wound.

